# Changes to Material Phase and Morphology Due to High-Level Molybdenum Doping of ZnO Nanorods: Influence on Luminescence and Defects

**DOI:** 10.3390/ma16093294

**Published:** 2023-04-22

**Authors:** Maksym Buryi, Vladimir Babin, Neda Neykova, Yu-Min Wang, Zdeněk Remeš, Katarína Ridzoňová, Filip Dominec, Marina Davydova, Jan Drahokoupil, Sergii Chertopalov, Lucie Landová, Ognen Pop-Georgievski

**Affiliations:** 1 FZU—Institute of Physics of the Czech Academy of Sciences, Na Slovance 1999/2, 182 21 Prague, Czech Republic; 2 Faculty of Nuclear Sciences and Physical Engineering, Czech Technical University in Prague, Trojanova 13, 120 00 Prague, Czech Republic; 3 Faculty of Electrical Engineering, Czech Technical University in Prague, Technická 2, 166 27 Prague, Czech Republic; 4 Department of Chemistry and Physics of Surfaces and Interfaces, Institute of Macromolecular Chemistry, Czech Academy of Sciences, Heyrovský sq. 2, 162 06 Prague, Czech Republic; 5 Faculty of Mathematics and Physics, Institute of Physics, Charles University, Ke Karlovu 5, 121 16 Prague, Czech Republic

**Keywords:** ZnO nanorods, molybdenum doping, morphology, luminescence, electron paramagnetic resonance

## Abstract

The influence of Mo on the electronic states and crystalline structure, as well as morphology, phase composition, luminescence, and defects in ZnO rods grown as free-standing nanoparticles, was studied using a variety of experimental techniques. Mo has almost no influence on the luminescence of the grown ZnO particles, whereas shallow donors are strongly affected in ZnO rods. Annealing in air causes exciton and defect-related bands to drop upon Mo doping level. The increase of the Mo doping level from 20 to 30% leads to the creation of dominating molybdates. This leads to a concomitant drop in the number of formed ZnO nanorods.

## 1. Introduction

Zinc oxide (ZnO) is a well-known optically active substance, typically appearing as bulk crystals, thin films, and nanoparticles [1,2,3], with a wide range of applications, including medicine [4], in particular, drug delivery [5], and wound scaffolding [6]. Another niche implementation is in scintillators. The well-known representatives are ZnO:Cu as the material for cathodoluminescence screens [7], whereas ZnO:Ga has the potential to be applied in alpha particle scintillation screens [8]. There are also common applications such as photocatalysis, electrocatalysis, gas or biological substances sensing, and Li-ion batteries [9,10,11,12,13]. Due to its strong photocatalytic properties, ZnO is also suitable for waste solidification/stabilization [14,15]. Historically, ZnO nanoparticles have been used in optoelectronic devices [16]. Since ZnO nanoparticles possess ultrafast excitonic luminescence (strongly below 1 ns), having a maximum of around 380 nm [17,18,19], another implementation of ZnO is the time of flight positron emission tomography [20]. Typically, ZnO nanorods are hexagonal and Wurtzite-like [21]. The free-standing particles, which are most commonly grown on the random nucleation seeds (NRPs), are considered in [17,22,23]. In most cases, the hydrothermal method, the simplest and cheapest one, is used [24,25]. There are several works dedicated to free-standing hydrothermally grown ZnO:Mo nanorods [23,26,27,28]. In particular, excitonic emission was found to be very sensitive to plasma treatment, X-ray irradiation, and annealing in air [23,27,29]. It was especially improved by Mo doping after annealing in air at 350 °C or after a hydrogen plasma treatment [23,27,30]. X-ray irradiation or oxygen plasma treatment suppresses the excitonic luminescence [29,31]. It should be noted that the shallow donors (SD) are influenced by the X-ray irradiation plasma treatment or annealing, as well [23,27,29]. The corresponding EPR signal is typically observed at the g factor *g* ≈ 1.95–1.96 in the ZnO nano-, micro-, or macrostructures (see, e.g., [23] and the references therein). Its origin was proposed to be Zn^+^ + D (D = Al, Ga, H) [23]. All of these findings were discovered in the low-level Mo-doped (not higher than 1%) free-standing ZnO nano- and microrods. The high-level Mo doping had a very different influence. To the best of our knowledge, there is only one report on high-level-doped ZnO [28]. In the report, only the ZnO:Mo(2%) sample demonstrated the ZnO hexagonal Wurtzite phase presence. The rest of the ZnO:Mo(5–25%) samples were a mix of the Zn_5_Mo_2_O_11_∙5H_2_O, MoO_3_∙2ZnO∙H_2_O, 2MoO_3_∙3ZnO∙H_2_O material phases. ZnO was never detected there [28]. However, in the present case, the dominating ZnO phase was detected in the ZnO:Mo(1, 5, 10%) samples. Moreover, even in the minority phase, the ZnO nanorods were detected in the ZnO:Mo(20%). The dominant material phase in the ZnO:Mo(20 and 30%) samples was Zn_5_Mo_2_O_11_∙5H_2_O. Therefore, one may conclude that the growth conditions strongly influence the Mo incorporation as well as the zinc molybdate phases-based creation. Therefore, the aim of the present work is to provide an extended experimental study of Mo incorporation in the ZnO:Mo rods as well as morphology and material phase change as a function of Mo doping level. This will reveal the role of the Mo-based random nucleation seeds on luminescent and scintillation properties, including ultrafast kinetics.

## 2. Experimental Techniques and Conditions

### 2.1. Samples Preparation

ZnO:Mo rods as nano- and micropowder with different Mo doping levels (1, 5, 10, 20, 30 wt. %) were grown using the hydrothermal method. The nutrient solution was prepared using 25 mM of zinc nitrate hexahydrate (Zn(NO_3_)_2_⋅6H_2_O) + ammonium heptamolybdate tetrahydrate (NH_4_)_6_Mo_7_O_24_⋅4H_2_O (NHMO) and 25 mM of hexamethylenetetramine (HMTA, C_6_H_12_N_4_). The temperature (90 °C) and time period (3 h) of growth were kept constant for all of the samples. After reaction termination, the powder precipitate was collected and purified by re-suspending it 3 times in water (200 mL) to remove any remaining non-reacted chemicals from the feed. Finally, the obtained suspension dried naturally through evaporation. The synthesis yield did not exceed 15 wt. % for all samples. For more details, see, e.g., [32]. 

### 2.2. Experimental Techniques Used for the Characterization of Samples

The crystalline structure of powders was characterized using an X-ray diffractometer (Empyrean, Malvern Panalytical, Almelo, The Netherlands) with Cu K_<α>_ radiation (*λ* = 1.54151 Å, at *U* = 45 kV, *I* = 30 mA). The X-ray diffraction (XRD) patterns were measured in the range 2*θ* from 5 to 120 degrees with a step of 0.026°. The characterization of powders was made using Bragg-Brentano geometry. The diffraction patterns were processed with the Rietveld Refinement program Topas 3 to perform fast sequential and parametric whole powder profile refinement of in situ time-resolved powder diffraction data [33]. The phase composition was given by structure fit, where intensities of peaks are calculated on the basis of atomic positions in the unit cell (the atomic positions were not refined). 

Raman analysis has been performed at room temperature using a blue laser with the wavelength *λ* = 488 nm, 50X × Olympus objective, and a grating of 2400 L/mm.

X-ray photoelectron spectrometry (XPS) measurements were performed on ZnO:Mo NRP samples using a K-Alpha^+^ XPS spectrometer (ThermoFisher Scientific, Horsham, UK) operating at a base pressure of 1.0 × 10^−7^ Pa [34,35]. The data acquisition and processing were performed using Thermo Avantage software. High-energy resolution core level spectra were measured using microfocused, monochromate Al Kα X-ray radiation (spot size of 400 µm, pass energies of 150 and 50 eV for survey and high-resolution measurements, respectively). During analysis, an incorporated charge compensation system using electrons and low-energy argon ions to prevent localized charge build-up was employed. All reported XPS spectra are averages of 10 individual measurements. The spectra were referenced to the C 1s peak of hydrocarbons at a binding energy of 285.0 eV controlled using photoelectron peaks of PET and metallic Cu, Ag, and Au standards. The atomic concentrations of the different chemical moieties were determined from the respective photoelectron peak areas of levels Si 2p, Mo 3d, C 1s, O 1s, and Zn 2p high-resolution spectra after modifying Shirley’s inelastic background subtraction. Assuming a simple model of a semi-infinite solid of homogeneous composition, the peak areas were corrected for the photoelectric cross-sections, the inelastic mean free paths of the electrons in question, and the transmission function of the spectrometer used. All spectra with high resolutions were fitted using Voigt profiles. The obtained quantitative XPS results report the average values and respective errors taken as standard deviation values from 8 independent measurements. The size and morphology of ZnO rods have been checked by scanning electron microscopy method (SEM) using an MAIA3, TESCAN electron microscope with the in-beam SE detector placed in the objective lens, and the electron beam energy set to 5 keV.

Cathodoluminescence (CL) was measured on a home-made spectrometer consisting of a parabolic mirror focusing the produced light onto a waveguide further propagating through a single-grating monochromator and photomultiplier tube H7711-13 to create CL images or Avaspec ULS2048LTEC spectrometer to record spectra. Cathodoluminescence measurements were spatially correlated with the energy dispersive X-ray spectroscopy (EDX) on a scanning electron microscope XL30ESEM with an installed EDX detector. In both cases of EDX and CL measurements, an acceleration voltage of 5 kV was used.

The steady-state photoluminescence (PL) spectra were excited by pulsed, optically filtered 1 mW UV LED at the wavelength band 340 ± 10 nm and measured in the 350–800 nm spectral range with 1 nm spectral resolution using the spectrally calibrated double grating monochromator SPEX 1672, long-pass filters (LP350 and LP600), a red sensitive photomultiplier, current preamplifier (gain 10 µA/V) and a lock-in amplifier referenced to the UV LED frequency 333 Hz. All PL spectra were divided by the spectral efficiency of the spectrometer, converted from wavelength to energy scale taking into account the Jacobian correction [36], and normalized at the wavelength 355 nm on the same value dominated by optical scattering of excitation light. After annealing, subsequent PL spectra were measured with 5 mg ZnO powder pressed in a Suprasil glass tube with an inner (and outer) diameter of 2 mm (and 3 mm, respectively), featuring a low fluorescence background. Low-temperature PL measurements were carried out with a 5 mg powder pressed into a disk pellet with a diameter 3 mm and glued together with double-sided conductive tape on a 10 × 10 × 0.3 mm^3^ Cu substrate in a closed He-cycle Oxford Instruments OptistatDry BLV cryostat (4–300 K).

EPR measurements were performed with a commercial Bruker EMXplus spectrometer in the X-band (9.4 GHz) within the 4–296 K temperature range using Oxford Instruments ESR900 cryostat. The sample was placed into the quartz tube. Spectra simulations were carried out in a “Easyspin toolbox 5.2.35” [37].

Annealing in air was carried out in a modular vertical tube furnace Tersid Carbolite with the possibility to elevate temperature up to 1000 °C.

## 3. Results and Discussion

### 3.1. Identification of Material Phases and Morphological Analyses

#### 3.1.1. Morphology by SEM

**Figure 1:**
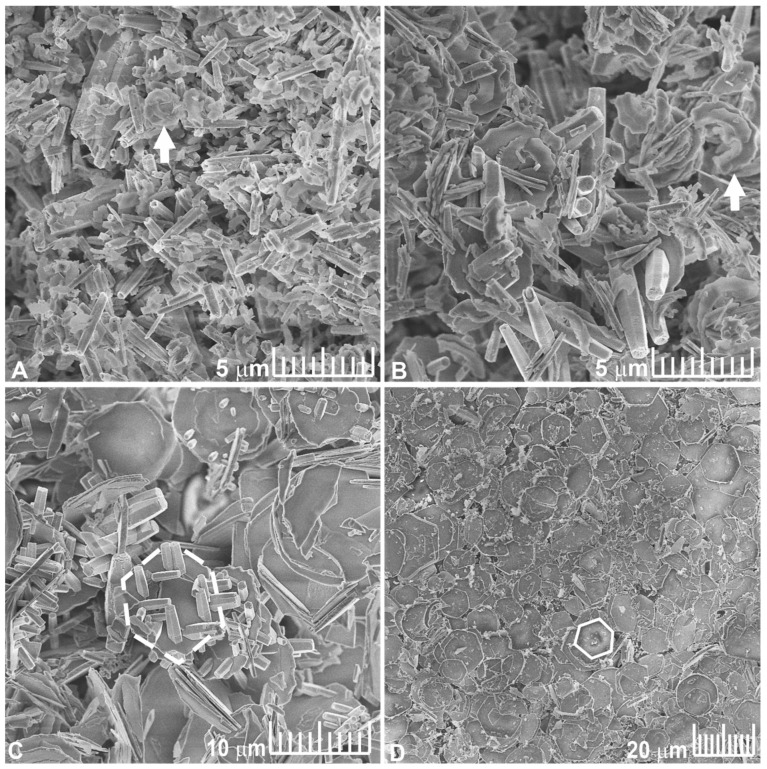
SEM images of ZnO:Mo(1%) (**A**), ZnO:Mo(5%) (**B**), ZnO:Mo(10%) (**C**), ZnO:Mo(20%) (**D**) NRP. Arrows stress the crescent-like structures. White hollow hexagons (dashed and solid) indicate the examples of flat hexagonal shape micro platelets.

SEM images of the ZnO:Mo NRP are shown in Figure 1. 

The ZnO:Mo(1%) NRP sample consisted of two types of hexagonal rods and greatly outnumbered crescent-like structures that were about 2.5 μm large (Figure 1A). The majority of the ZnO:Mo(1%) NRP is, in general, represented by the roughly 500-nm-thick and 2–5-μm-long rods. The rest of the rods are about 3 μm thick and about 5 μm long.

The ZnO:Mo(5%) NRP samples consisted of hexagonal rods with a thickness of about 1 μm and a length over 5 μm and crescent-like structures about 5 μm large (Figure 1B). The size of the latter increased significantly in the ZnO:Mo(5%) NRP sample compared to the ZnO:Mo(1%) NRP, where crescent-like structures are rare.

The ZnO:Mo(10%) NRP sample is represented by the large rods (about 1.5 μm thick and 5–7 μm long) and hexagonal platelets with a diameter of about 10 μm (Figure 1C). The number of micro platelets was significantly higher than the number of rods. The rods seem to grow on the surface of the micro platelets, as well (Figure 1C).

Large amounts of nucleation centers provided by Mo doping hovering in the solution of the NRP influence the rods’ growth. The ZnO:Mo(20 and 30%) NRP samples consisted of hexagonal platelets with a diameter of about 10 μm, as can be seen in the example of the ZnO:Mo(20%) (Figure 1D). 

#### 3.1.2. Phase Composition by XRD

**Figure 2:**
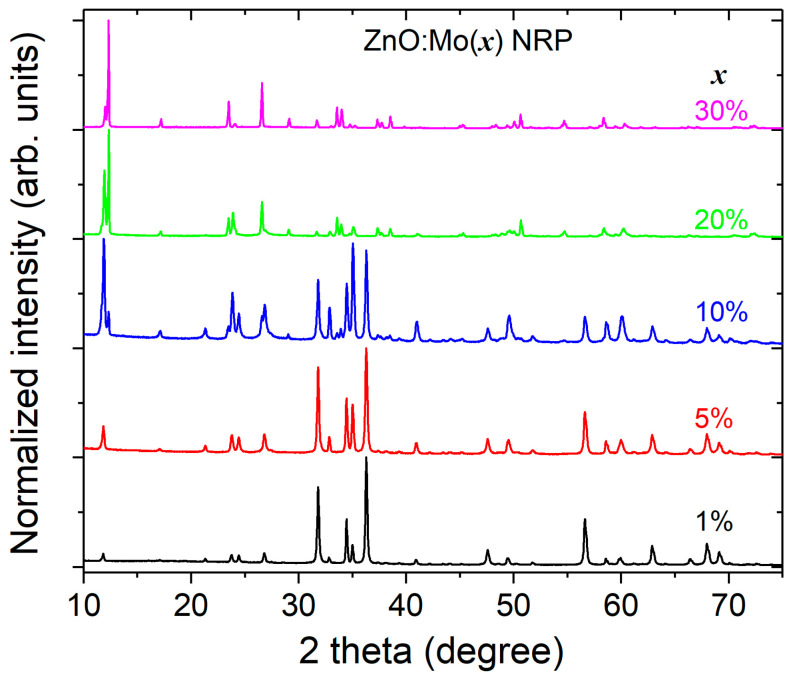
The XRD patterns of ZnO:Mo(x) NRP samples, x = 1, 5, 10, 20 and 30% (2theta-theta mode).

The XRD pattern of the ZnO:Mo(1, 5, 10, 20, 30%) NRP samples is shown in Figure 2. The ZnO:Mo(1, 5, 10%) NRP samples have a hexagonal Zincite phase (PDF 00-005-0664). Increasing Mo concentrations led to a new phase formation (monoclinic, C_12_H_7_NO_2_, PDF 00-034-1749), as can be seen in Figure 2. The length of this phase increases with Mo concentration, and reaches its maximal value at 10%. Additionally, starting from a 10 wt. % Mo doping level, a hexagonal phase (Zn_5_Mo_2_O_11_·5H_2_O, PDF 00-030-1486) is formed. It increases at 20 and 30% Mo doping level and reaches a significantly higher level in the ZnO:Mo(30%) NRP. Moreover, the weak reflections of d-MoN hexagonal phase (MoN, PDF 04-014-2477) were detected, as well. The PDF patterns for all of the material phases are shown in Figure S1 in the Supplementary Information section, where they are shown along with the XRD pattern of the ZnO:Mo(10%) NRP sample. Both C_12_H_7_NO_2_ and the d-MoN phases expectedly occur as degraded precursors used for the NRP samples grow (see Experimental). This also has some correlation with the Raman measurements below. The presence of ZnO, as well as the d-MoN phase, was not confirmed in the ZnO:Mo(20 and 30%) NRP samples. These observations lead to the conclusion that the nano- and microrod structures in Figure 1A–C originate from ZnO, whereas the hexagonal platelets in Figure 1C,D must be created by the Zn_5_Mo_2_O_11_·5H_2_O. The morphology of the d-MoN phase is not known. The seeding layer must afford additional space for the Mo-based nucleation seeds to be placed on the surface of the layer.

Molybdenum appearing in the ZnO host changes its charge state from Mo^6+^ existing in the precursor ((NH_4_)_6_Mo_7_O_24_·4H_2_O) to Mo^4+^, as confirmed by the present work as well as discussed in previous works [23,27]. This is a sign of reduction, indicating processes that could be connected to the redox reaction existence. At the same time, HMTA (C_6_H_12_N_4_) is degraded by the removal of hydrogen and attachment of oxygen, which is a typical redox reaction in organic chemistry [38]. Considering the improved presence of naphthalimide in the ZnO:Mo samples upon the increased doping level of Mo, the Mo precursor can be expected to be a catalysator in the redox reaction discussed. The tentative and simplest example of the reaction can be given as follows:

2C_6_H_12_N_4_ + 2O^2−^ + 4h^+^ ((NH_4_)_6_Mo_7_O_24_·4H_2_O, catalysis) → C_12_H_7_NO_2_ + 7H_2_ + 3N_2_ + NH_3_ (1)

In the NRP samples with Mo doping levels of 20–30%, the concentration of molybdenum precursor in the solution is very high, leading to the chemical reactions resulting in the creation of zinc molybdates (hexagonal platelets), as confirmed by XRD (Figure 2). Obviously, the zinc molybdate phases were grown first (the corresponding chemical reaction rates should be very fast) in the ZnO:Mo(20%) NRP sample, strongly lowering the concentration of Mo in the solution.

#### 3.1.3. Raman Spectroscopy

**Figure 3:**
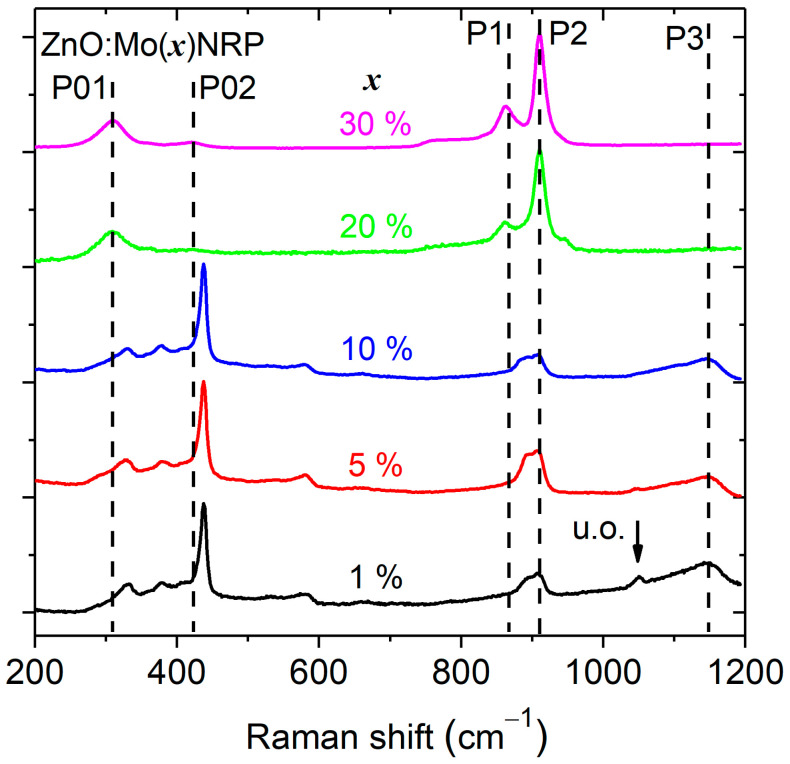
Raman spectra of the ZnO:Mo(x) NRP, x = 1, 5, 10, 20, and 30%. P01,02,1,2,3 peaks are attributed to the material phases different than ZnO. “u.o.” indicates peaks originating from unknown material phase.

Figure 3 shows the Raman spectroscopy results for the Mo-doped ZnO NRP with different doping concentrations (1, 5, 10, 20, and 30 wt. %).

The spectra are composed of five prominent peaks located at about 331, 438, 580, 862, and 910 cm^−1^. All Raman spectra show the *E*_2_ (high) mode located at 438 cm^−1^, which corresponds to the high-frequency optical phonon mode of the ZnO wurtzite crystal structure [39,40]. The intensity of the *E*_2_ (high) mode is the most intense in the Raman spectra for Mo doping levels ranging from 1 up to 30 wt. %, which is a sign of a well-crystallized ZnO phase. The *E*_2_ (high) mode becomes weaker and broader at high Mo doping levels (20 and 30 wt. % of Mo), indicating a defective or small domain size in the ZnO phase. The peak centered at 331 cm^−1^ is attributed to the second-order mode (*E*_2_ low) [39]. 

The broad peak at 580 cm^−1^, which corresponds to normally Raman inactive *E*_1_ (LO) mode and is attributed to oxygen vacancies and zinc interstitial [41,42], is present at low Mo doping levels and not visible at high Mo doping levels. 

There were five more peaks observed near 309 cm^−1^ (P01 in Figure 3), 423 cm^−1^ (P02 in Figure 3), 862 cm^−1^ (P1 in Figure 3), 910 cm^−1^ (P2 in Figure 3), and 1149 cm^−1^ (P3 in Figure 3). The intensity of the P01,02,1,2 peaks slowly increases with Mo doping level and becomes predominant at high Mo doping levels (20 and 30 wt. % of Mo). This correlates perfectly well with the increase of the Zn_5_Mo_2_O_11_·5H_2_O phase presence in the corresponding XRD patterns (Figure 2). The P3 peak is very broad, which indicates multiple contributions, and its intensity increases very little with the Mo content in the Raman spectra of the ZnO:Mo(1, 5, 10%) samples. The peak P3 is almost totally absent in the spectrum of the ZnO:Mo(20%), and it is totally absent in the spectrum of the ZnO:Mo(30%). This has a good correlation with the XRD pattern corresponding to the d-MoN phase as a consequence of the ZnO:Mo(1, 5, 10, 20, 30%), as can be seen in Figure 2 and Figure S1 in the Supplementary Information section. Therefore, the P3 peak has been attributed to the d-MoN phase. 

#### 3.1.4. Surface Composition by XPS

XPS spectroscopy was used to probe the chemical structure of the ZnO:Mo NRP samples. 

**Figure 4:**
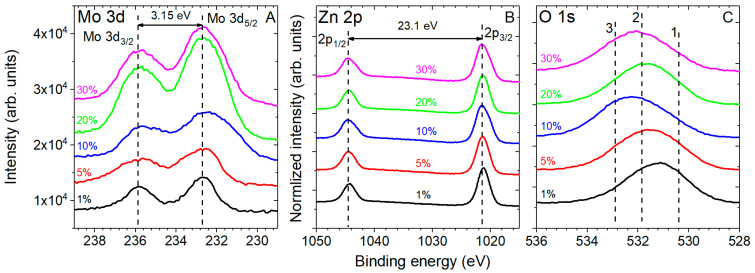
High-resolution XPS spectra of ZnO:Mo NRP samples acquired in the Mo 3d (**A**), Zn 2p (**B**), and O 1s (**C**) regions. Individual spectra were vertically stacked for better representation. Numbers 1–3 indicate the specific peaks produced by: 1—Zn-O-Zn; 2—Zn-O-H, Zn-O^−^, C=O, Si-O, Zn-O-Mo; 3—C-O (see also Table 1). The percentage values correspond to the Mo doping level.

Typical XPS high-resolution Mo 3d, Zn 2p, and O 1s spectra of different structures measured in ZnO:Mo NRP samples are shown in Figure 4.

The high-resolution XPS measurements in the Mo 3d region (Figure 4A) verified the presence of molybdenum spin–split doublet with a Mo 3d_5/2_ peak centered at about 233 eV and a Mo 3d_3/2_ peak showing 3.15 eV separation from the main contribution. The FWHM is also different (the Mo 3d_5/2_ is considered) for different Mo doping levels: 2.52 eV in the ZnO:Mo(5%) NRP, 3.14 eV in the ZnO:Mo(10%) NRP, 2.7 eV in the ZnO:Mo(20%) NRP, and 2.51 eV in the ZnO:Mo(30%) NRP. The specific trend shown by the FWHM values in the differently doped ZnO:Mo NRP is due to the presence of several Mo-containing material phases. The domination of the zinc molybdates is getting stronger upon the Mo doping level, and as a sequence, the broadening of the Mo 3d peaks in the ZnO:Mo(5 and 10%) NRP is observed. Further-increased Mo doping levels (ZnO:Mo(20 and 30%) NRP samples) led to the narrowing of the Mo 3d peaks due to the fast thinning of the ZnO phase and the creation of zinc molybdates.

All ZnO:Mo NRP samples are characterized by a spin–split doublet with a Zn 2p_3/2_ peak centered at about 1022.1 eV and a Zn 2p_1/2_ peak showing 23.0 eV separation from the main contribution (Figure 4B), as reported in previous work [40]. They are broadening with the increase of the Mo doping level (Figure 4B). Therefore, one may expect them to appear as a number of contributions from different material phases discovered by XRD (Figure 2 and Figure S1 in the Supplementary Information section).

The O 1s spectrum of all materials was characterized by three contributions arising from lattice oxygen (Zn-O-Zn), non-lattice oxygen (Zn-O-H, Zn-O^−^, C=O, Si-O), and C-O contributions centered at about 530.5 (peak 1 in Figure 4C), 531.9 (peak 2 in Figure 4C) and 532.9 eV (peak 3 in Figure 4C), respectively [40,43]. The O=C and C-O-H contributions likely originate from the remnants of the precursor adsorbed on ZnO surfaces. The peak corresponding to Zn-O-Mo bonds should strongly overlap with the non-lattice oxygen moieties and uncovered substrate Si-O signals at 531.9 eV. Therefore, the exact quantification in this case could not be performed with high reliability.

Possible N 1s contributions arising from the precursors during synthesis, ammonium molybdate tetrahydrate, and zinc nitrate hexahydrate were below the detection limits of the XPS measurements, further pointing to the incorporation of the molybdenum into the NC structure. 

All of the experimental spectra in Figure 4 were fitted with the calculated ones (standard Gaussian shapes). XPS regions, as well as chemical moieties and their weight fractions, are listed in Table 1.

Based on the XPS data, molybdenum ions present on the NRP samples surface are Mo^6+^. Remarkably, their content increases upon the Mo doping level until the ZnO:Mo(30%) NRP. There, the Mo^6+^ content is a bit lower than in the ZnO:Mo(20%) NRP sample, probably, this is the consequence of the decreased effective surface area in the ZnO:Mo(30%) NRP sample due to the hexagonal platelet presence (Zn_5_Mo_2_O_11_·5H_2_O) (see Figure 2 and Figure S1 in the Supplementary Information section). Note that the Mo/Zn ratio is the same in the ZnO:Mo(20 and 30%) NRP (Table 1).

Carbon on the surface of the ZnO:Mo NRP samples originates from the remained precursors adsorbed on the ZnO surfaces. Its overall content is about 25–35% in the ZnO:Mo NRP samples.

Overall oxygen content increases in the ZnO:Mo(1, 5 and 10%) NRP while it was decreased by 0.7 at.% in the ZnO:Mo(20%) NRP as compared to the ZnO:Mo(10%) NRP sample (Table 1). It was decreased by 3.5 at.% in the ZnO:Mo(30%) NRP as compared to the ZnO:Mo(10%) NRP sample (Table 1). This also correlates well with the Mo^6+^ content. This is the result of greater carbon content in the ZnO:Mo(30%) NRP compared to the ZnO:Mo(10%) NRP samples covering the area of the ZnO:Mo(30%) NRP sample (Figure 1D). Therefore, the increase, namely of the Zn-O-Mo moiety content, can be expected in this case. No specific trends were observed for the single moieties. This is the result of the interplay between different oxygen-containing moieties, as can be seen in Table 1. 

The overall content of Zn^2+^ remained almost the same in the ZnO:Mo(1 and 5%) NRP, while it gradually decreased in the ZnO:Mo(10, 20, and 30%) NRP samples (Table 1). The Mo/Zn content ratio monotonously increased upon the Mo doping level (Table 1). This has a good correlation with the XRD and Raman measurements above, indicating the escalating presence of the Mo-containing material phases in the NRP samples (Figure 2 and Figure 3).

### 3.2. Photoluminescence Properties

**Figure 5:**
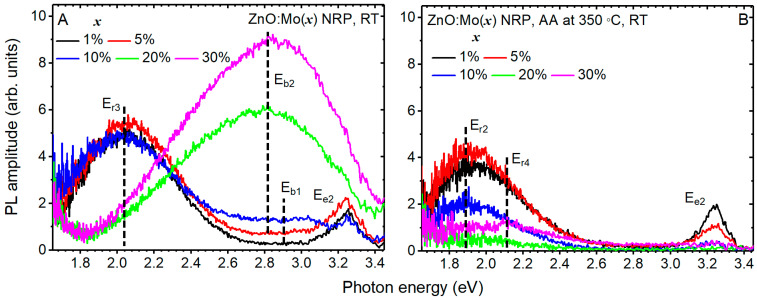
PL spectra measured at RT before (**A**) and after the annealing in air (AA) at 350 °C (**B**) in ZnO:Mo(x), x = 0.05, 0.25, 1, 5, 10, 20, and 30% NRP samples, respectively. E_e1,2_, E_r1–4_ and E_b1,2_ stress specific emission bands.

The photoluminescence of hydrothermally grown ZnO nano- and microrods is sensitive to annealing in air, with the strongest effect on the exciton band observed for the annealing temperature of 350 °C [17,22,26,31]. Therefore, the influence of annealing in air at 350 °C was studied in the present case. The PL spectra of the as grown NRP samples are shown in Figure 5A. They were composed of two–three bands: E_r3_ (2.04 eV) and E_e2_ (3.25 eV) in ZnO:Mo(1%) NRP; E_r3_ (2.04 eV), E_e2_ (3.25 eV) and E_2_ (very broad, having maximum at about 2.8 eV) in ZnO:Mo(5 and 10%) NRP; E_b2_ (very broad, having maximum at about 2.8 eV) in ZnO:Mo(20 and 30%) NRP. Based on previous works [27,29,31,44,45], the E_r3_ and E_e2_ bands were attributed to neutral zinc vacancy-based defects (in particular, they are of the two-component origin ascribed to neutral zinc vacancy-based defects (V_Zn_^0^ and V_Zn_^0^ + D, D is some defect) [22]) and excitons [46,47], respectively. Mo has almost no effect on the E_r3_ and E_e2_ bands in the ZnO:Mo(1, 5, 10%) NRP (see Figure 5A). This also differs from the tendencies previously reported for free-standing ZnO:Mo nanorods [23]. Based on Figure 1 and the XRD analysis above, the ZnO:Mo(1, 5, and 10%) NRP consists of ZnO, as well as zinc molybdate-based phases. The ZnO phase is thinning upon the increased Mo doping level (see Figure 2). Therefore, the luminescence is expected to fade. The fact that it does not vanish but remains at the same intensity level (see Figure 5A) provides evidence for the improved luminescence properties with the presence of Mo. Based on these considerations, one can expect the strongest E_r3_ and E_e2_ bands to appear in the PL spectra of ZnO:Mo(5%) NRP (Figure 5A).

The E_b2_ band (Figure 5A) is expected to originate from Mo-O-like emission centers in zinc molybdate-based phases (Mo^6+^-O^2−^ → Mo^5+^-O^−^ known in ZnMoO_4_ [48,49]).

Low blue photoluminescence background, marked as the E_b1_ band in Figure 5, is sometimes observed due to the contamination of the sample by organic dust particles/remained precursors. It can be minimized by the careful selection of the area illuminated by the excitation light. This explains why this band is absent in the PL spectra of the ZnO:Mo(1%) NRP sample.

The PL spectra of the NRP samples annealed in air at 350 °C were composed only of one–three bands: E_r2_ and E_e2_ in the ZnO:Mo(1, 5, 10%) NRP; E_r2_ in the ZnO:Mo(20%) NRP; E_r2,4_ in the ZnO:Mo(30%) NRP. The E_r2_ band remained almost unchanged in the annealed ZnO:Mo(1, 5%) NRP. This is consistent with the behavior of the E_r3_ band in the as grown ZnO:Mo(1, 5%) NRP samples discussed above (Figure 5). Therefore, the same effect of Mo can be expected there. The intensity of the E_r2_ band measured in the annealed ZnO:Mo(10%) NRP is lower by a factor of ~2 compared to the annealed ZnO:Mo(1, 5%) NRP samples. This can be explained by the creation of Mo^5+^ (not observed in the annealed ZnO:Mo(1%) NRP discussed in detail below). The Mo^5+^ must be created from Mo^4+^ by the hole capture, as discussed in previous works [23,26]. The hole is transferred from the neutral zinc vacancy responsible for the E_r2_ [23], and, therefore, the E_r2_ decreased. Negligibly weak E_r2_ band has been measured in the annealed ZnO:Mo(20, 30%) NRP. The negligibly weak E_r4_ band at roughly 2.11 eV was resolved in the PL spectrum of the annealed ZnO:Mo(30%) NRP. It corresponds very well to one of the components (at about 2.1 eV) in the complex 2.0 eV emission band observed in the ZnO:Mo free-standing nanorods [22,23,27,29,31]. It is noteworthy that the ZnO phase is minor while the presence of zinc molybdates is strong in the ZnO:Mo(20, 30%) NRP (see XRD results above). The intensity of the red PL bands (E_r2–4_ in Figure 5) in the NRP samples was not enough to provide reliable statistics, and, as a result, the precise analysis of the corresponding decay kinetics was impossible.

The E_b2_ band was not observed in the annealed ZnO:Mo(20, 30%) NRP samples (Figure 5B). All of these allowed us to conclude that annealing in air has the tendency to improve the ZnO structure by removing surface defect states and causing the partial decomposition of zinc molybdates [26].

The E_e2_ band intensity was strongly lowered upon the increased Mo doping level from 1 to 5 and 10% in the NRP samples (Figure 5A). This should be the effect of the dominating presence of the unintentional zinc molybdate (Zn_5_Mo_2_O_11_·5H_2_O) and d-MoN phases (Figure 2 and Figure S1 in the Supplementary Information section). The Mo-O-like emission (E_b2_ band in Figure 5A) has most likely been transformed into a non-radiative path due to annealing (the Mo charge state is changed according to the EPR below), and the Zn_5_Mo_2_O_11_·5H_2_O is now reabsorbing the high emission energy of the exciton emission centers in the ZnO phase. No exciton band was detected in the annealed ZnO:Mo(20, 30%) NRP. This is expected since there was no ZnO phase, and the corresponding exciton emission was detected in the as grown ZnO:Mo(20, 30%) NRP samples.

**Figure 6:**
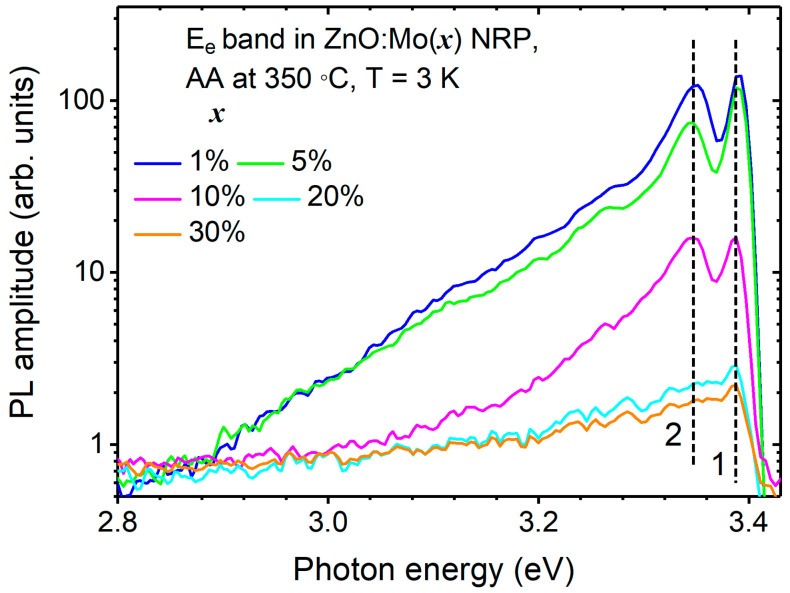
UV PL spectra of the E_e_ band measured at 3 K in ZnO:Mo(x) NRP (B) samples, x = 1, 5, 10, 20, 30%. Numbers 1, 2 stress specific exciton-related transitions.

To study the E_e2_ band in more detail, the PL spectra of the ZnO:Mo(1, 5, 10, 20, and 30%) NRP samples annealed in air at 350 °C were measured at 3 K, as well, and shown in Figure 6.

The E_e2_ band is characterized by a free exciton peak at 3.39 eV and a relatively asymmetric shoulder at lower energies. The bound exciton emission at 3.35 eV (peak 2) was attributed to the neutral-donor-bound exciton complexes [50,51] that may originate from surface-related defects, such as V_Zn_^0^, which act as a neutral acceptor. The 2.9–3.3 eV shoulder is typical for LO phonon replicas, two-electron satellites, and donor–acceptor pairs [47]. The E_e2_ band in the ZnO:Mo(20 and 30%) NRP was negligibly weak, but still, peak 1 could be resolved, proving the existence of a very brief ZnO phase.

### 3.3. CL and EDX Mapping

**Figure 7:**
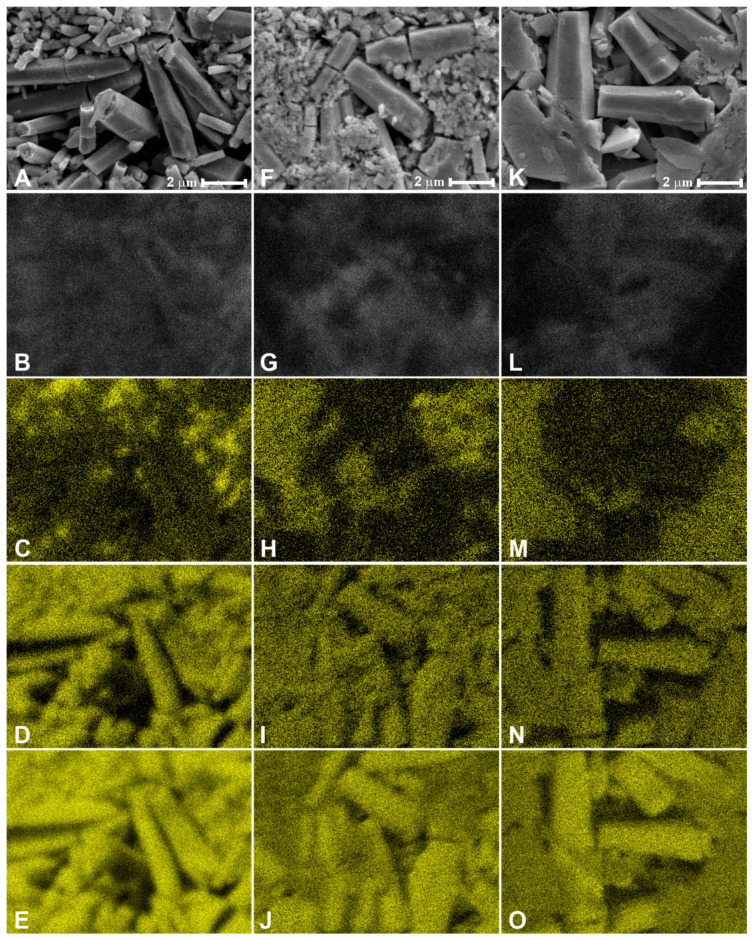
SEM images (**A**,**F**,**K**), CL (**B**,**G**,**L**) and EDX maps of Mo (**C**,**H**,**M**), Zn (**D**,**I**,**N**) and O (**E**,**J**,**O**) measured in: ZnO:Mo(1%) NRP (**A**–**E**); ZnO:Mo(5%) NRP (**F**–**J**); ZnO:Mo(10%) NRP (**K**–**O**).

To study the spatial distribution of luminescence over the nanorods and the influence of Mo on it, the correlated SEM, spectrally unresolved CL, and EDX images were obtained for the NRP samples. They are shown in Figure 7. Smaller rods emitted brighter than the larger ones in the ZnO:Mo(1%) NRP sample (Figure 7A,B). This also correlates with the Mo spatial distribution (Figure 7C), i.e., Mo tends to stay in the smaller ZnO rods. Partly, a similar conclusion can be made in the case of the ZnO:Mo(5%) NRP sample (Figure 7F–H). However, in this case, larger rods also emit brightly. The situation is completely different in the ZnO:Mo(10%) NRP (Figure 7K–M). There, the luminescence originates exclusively from the large rods, whereas Mo is concentrated in the platelet structures.

All of these observations have a very good correlation with the PL measurements above and confirm the influence of Mo on the luminescence properties of the ZnO:Mo nanorods, as well as the transformation of ZnO:Mo into the complex zinc molybdate phase.

### 3.4. Core and Shell Shallow Donors as Well as Mo^5+^ Detected by EPR

**Figure 8:**
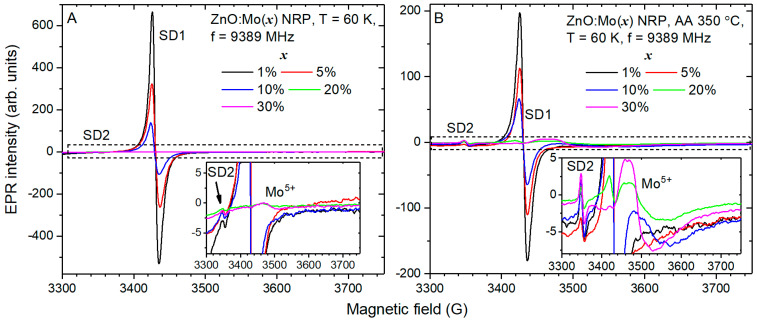
EPR spectra measured at 60 K before (**A**) and after annealing in air at 350 °C (**B**) in ZnO:Mo(x) NRP. SD1,2 indicate shallow donor signals from core and shell, respectively.

EPR spectra measured in the as grown ZnO:Mo NRP samples are shown in Figure 8.

The EPR spectra of the ZnO:Mo(1, 5, 10%) NRP were composed of the SD1,2 signals: at the *g* factor *g* = 1.954 (SD1), the typical signal of ZnO (see [22,27,29,31] and the references therein), and at *g* = 2.0023 (SD2), the *g* factor of the free electron [52]. The SD1 signal originates from the core (bulk part of a single ZnO nanorod), whereas the SD2 signal originates from the shell (the part of the ZnO nanorod at the surface) based on the core–shell model [53]. The SD1 signal is typical for ZnO and is produced by the shallow donor Zn^+^ + D, D = Al/Ga/H [54,55,56,57,58]. The intensity ratio for the SD1 signal in the ZnO:Mo(1, 5, 10%) NRP is 5:2:1. This can be explained as follows: the source of the SD1 signal captures a hole (D + Zn^+^ + h^+^ → D + Zn^2+^) or, oppositely, releases an electron (D + Zn^+^ → D + Zn^2+^ + e^−^). The deliberated electron is then captured by Mo^6+^, creating Mo^4+^ (Mo^6+^ + 2e^−^ → Mo^4+^), whose existence is proved by the creation of Mo^5+^ after annealing in air, as discussed below.

The SD2 signal is about two orders of magnitude weaker than the SD1 one (see Figure 8A). The intensity ratio for the SD2 signal in the ZnO:Mo(1, 5, 10%) NRP is 3:2:2.

There was a new, relatively broad signal at the *g* ~1.92 (typical for Mo^5+^ [23,27,29,59]) detected in the EPR spectra of the as grown ZnO:Mo(20 and 30%) NRP samples (Figure 8A). Since the correlation between the signal intensity and Mo content was observed, it was attributed to the Mo^5+^. To scrutinize its origin, the Mo^5+^ spectra have been fitted using Equation (2):

(2) $$\hat{H}=\beta \hat{S}\hat{g}H,$$

where β is the Bohr magneton, $\hat{S}$ is the vector of electron spin operator (electron spin S = 1/2 was considered), $\hat{g}$ is a g tensor, and *H* is the vector of the resonance magnetic field. The terms counting for hyperfine coupling with the ^95,97^Mo nuclei were omitted since the Mo^5+^ signals observed (Figure 8A) were broad and, as a sequence, the corresponding contributions to the spectra were not resolved (for more details, see previous works [23,52]). The experimental and calculated spectra are shown in Figure S2 in the Supplementary Information section for the as grown ZnO:Mo(20 and 30%) NRP samples. The fit is very good. The fit parameters are listed in Table 2.

The Mo^5+^ spectrum measured in the annealed ZnO:Mo(20 and 30%) NRP is, indeed, composed of two signals referred to as Mo1,2 in Table 2. The Mo1 is isotropic with *g* = 1.918. Its *g* factor differs from the Mo^5+^ signal detected in the as grown ZnO:Mo free-standing nanorods samples (*g* = 1.905 [23]). Another signal, Mo2, is anisotropic. The Mo1 is significantly more prevalent than the Mo2 component in the as grown ZnO:Mo(20%) NRP, with the intensity ratio being 7:1 (Mo1 to Mo2). However, the situation is inverse in the ZnO:Mo(30%) NRP, with the intensity ratio being 1:2 (Mo1 to Mo2) (see Table 2). The Mo1 intensity was increased by about 20 times in the ZnO:Mo(30%) NRP compared to the ZnO:Mo(20%) NRP. All of these allowed us to conclude that the Mo1,2 contributions originate from zinc molybdates and d-MoN (Figure 2 and Figure S1 in the Supplementary Information section) and not the ZnO phase in the as grown ZnO:Mo(20 and 30%) NRP samples.

### 3.5. NRP Samples Annealed in Air at 350 °C

Annealing in air at 350 °C resulted in a roughly threefold drop in the SD1 signal compared to the as grown samples (see Figure 8B). Its spectral position remained unchanged. This has previously been observed for the ZnO:Mo free-standing nanorods [23]. The intensity ratio for the SD1 signal in the ZnO:Mo(1, 5, 10, and 20%) NRP is 99:59:34:1. Remarkably, a very weak SD1 signal also appeared in the EPR spectrum of the annealed ZnO:Mo(20%) NRP; however, it was not observed in the as grown ZnO:Mo(30%) NRP. This may indicate the creation of the ZnO phase by the destruction of the zinc molybdate. Part 99:59:34 = 3:2:1 for the annealed ZnO:Mo(1, 5, 10%) NRP has approximately the same tendency as discussed above for the as grown ZnO:Mo(1, 5, 10%) NRP. Moreover, it is consistent with the trends observed for the E_e2_ band upon the Mo doping level (Figure 5B). The smaller the number of shallow donors, the smaller the number of free carriers, and, as a result, the exciton-related band drops.

The SD2 signal was increased by about 1.5–3 times in the ZnO:Mo NRP annealed in air at 350 °C compared to the as grown samples (see Figure 8B). The intensity ratio for the SD2 signal in the annealed ZnO:Mo(1, 5, 10, 20, and 30%) NRP is 2:1:2:1:2. This is a rather random ratio that is inconsistent with the trends observed for the as grown NRP samples (see Figure 8A). All of these indicate that the SD2 signal should originate from dangling bonds, which are obviously affected by oxygen from air refilling the oxygen vacancies initially existing in the materials.

Again, the relatively broad signal appeared at the *g* factor at roughly *g* = 1.9 (typical for Mo^5+^ [23,27,29,59]) in the EPR spectra of ZnO:Mo(10%) NRP and at the *g* factor at roughly *g* = 1.92 (also typical for Mo^5+^ [23,27,29,59]) in the ZnO:Mo(20 and 30%) NRP samples after annealing in air (Figure 8B). Similar to the as grown NRP samples, the *g* = 1.9 signal was attributed to the Mo^5+^ incorporated into the ZnO rods [23,26]. The *g* = 1.92 signal is expected to originate from zinc molybdates, considering the dominating zinc molybdates phase in the ZnO:Mo(20, 30%) NRP and the shift of the *g* factor value compared to the ZnO:Mo(10%) NRP. The number of the Mo^5+^ is larger in the annealed ZnO:Mo(30%) NRP as compared to the ZnO:Mo(20%) NRP. This could be explained by the larger amount of Mo^4+^ existing in the ZnO:Mo(30%) NRP compared to the ZnO:Mo(20%) NRP sample.

To gain better insight into the Mo distribution over different surroundings, the Mo^5+^ spectra detected in the annealed ZnO:Mo(10, 20, and 30%) NRP samples (Figure 8B) have been fitted using Equation (2). Again, the terms counting for the hyperfine coupling with the ^95,97^Mo nuclei were omitted since the Mo^5+^ signals observed (Figure 8B) were very broad, and, as a sequence, the corresponding contributions to the spectra were not resolved [23,52]. The calculated spectra fit the experimental ones very well, as can be seen in Figure S3 in the Supplementary Information section. The fit parameters are listed in Table 2, as well. The Mo^5+^ signal was composed of one Mo1′ component in the ZnO:Mo(10%) NRP, the superposition of Mo1′ and Mo2′ signals in the ZnO:Mo(20%) NRP, and the superposition of Mo2′ and Mo2″ signals in the ZnO:Mo(30%) NRP samples. The Mo1′ one is isotropic with the *g* = 1.908. It is close to the value reported earlier for the low-level doped ZnO:Mo(0.05–1%) [23]. The intensity of the Mo1′ component lowers upon Mo doping (the zinc molybdate and d-MoN phases are created). Therefore, it is expected to originate from ZnO rods. It should be noted that the ZnO nanorods presence was deduced from EPR spectra in Figure 8B in the ZnO:Mo(20%) NRP sample. It was not detected at all in the ZnO:Mo(30%) NRP sample. The ZnO:Mo(30%) NRP sample is composed exclusively of the hexagonal platelets of the zinc molybdate phase (see Figure 1D and Figure S1 in the Supplementary Information section). The Mo2′ has a slightly rhombic g tensor. It is about 6 times weaker than the Mo1′ one in the ZnO:Mo(20%) NRP. The intensity of the Mo2′ signal in the ZnO:Mo(30%) NRP was increased by roughly twice compared to the ZnO:Mo(20%) NRP. The new signal, Mo2″, also appears there, roughly three times more than the Mo2′ one. The g tensor values of the Mo2″ signal slightly differ from the Mo2′ signal. Therefore, both signals are expected to originate from two different Mo^5+^ centers localized in the zinc molybdate phase and d-MoN, most likely at the regular and perturbed Mo sites.

## 4. Conclusions

ZnO:Mo nanorods were grown as free-standing particles. At low doping levels (below 5%), Mo becomes incorporated into the ZnO rods bulk, whereas the increased Mo content from 5–10–30% led to the creation and dominance of the byproduct material phases, found to be Zn_5_Mo_2_O_11_·5H_2_O and d-MoN. The Zn_5_Mo_2_O_11_·5H_2_O phase is predominately present at the hexagonal platelets. The morphology of d-MoN is not known. XPS indicates the presence of Mo as Mo^6+^. The luminescence properties of the free-standing particles were as follows. Exciton emission is multicomponent. Besides the typical free and bound exciton components, the bound exciton emission at 3.35 eV was attributed to the neutral-donor-bound exciton complexes [50,51] that may originate from surface-related defects such as V_Zn_^0^, which acts as a neutral acceptor in the free-standing ZnO nanorods.

Shallow donor levels, whose presence was confirmed by EPR, are also affected by the Mo states. Moreover, the Mo^4+^ presence in the materials was confirmed, as well. Its content depends on the Mo doping level. It appears partly in the ZnO host and in the byproduct Mo-related material phases. Considering the much larger sensitivity of EPR compared to XPS (sensitive to the surface only), the mentioned quantity of Mo^4+^ appears far below the measurable limit of the XPS, while Mo^6+^ strongly dominates, taking into account the large doping levels of Mo.

## Figures and Tables

**Table 1:** Atomic fractions (at.%) of chemical moieties present on the surface of the ZnO:Mo NRP samples as determined by XPS.

XPS Region	Chemical Moieties	Binding Energy, eV	Mo Doping Level (at.%)				
			1.0	5.0	10.0	20.0	30.0
			Fraction of Moieties and Bonds (at.%)				
Mo 3d	Mo ^6+^	233.1 ± 0.3	1.6 ± 0.2	2.5 ± 0.4	5.3 ± 0.1	6.0 ± 0.1	5.4 ± 0.6
C 1s	C-C, C-H	285.0 ± 0.2	16.0 ± 1.6	11.5 ± 3.5	10.1 ± 0.8	15.8 ± 0.9	14.0 ± 1.0
	C-O	286.4 ± 0.2	4.5 ± 0.5	8.7 ± 2.6	12.1 ± 0.9	7.1 ± 0.4	13.7 ± 0.9
	C(=O)-O	289.0 ± 0.2	3.5 ± 0.4	3.4 ± 1.0	3.4 ± 0.3	2.8 ± 0.2	3.8 ± 1.0
	Total C	-	24	23.6	25.6	25.7	31.5
O 1s	Zn-O-Zn	530.6 ± 0.2	15.3 ± 0.3	14.4 ± 1.1	14.1 ± 0.3	15.8 ± 0.3	12.4 ± 0.8
	Zn-O-H, Zn-O^−^, C=O, Si-O Zn-O-Mo	531.8 ± 0.3	25.2 ± 0.5	22.7 ± 1.7	22.9 ± 0.5	24.7 ± 0.4	20.7 ± 2.4
	C-O	533.1 ± 0.2	8.6 ± 0.2	12.9 ± 1.0	13.7 ± 0.4	9.8 ± 0.2	14.4 ± 1.7
	Total O	-	49.1	50	51	50.3	47.5
Zn 2p_3/2_	Zn ^2+^	1022.2 ± 0.4	25.3 ± 1.5	23.8 ± 3.1	18.5 ± 0.8	17.9 ± 0.7	15.8 ± 2.7
Mo/Zn			0.06	0.11	0.29	0.34	0.34

**Table 2:** The g tensor values, peak-to-peak width (ΔH_pp_), and double integral intensity (I) are determined from fit for the EPR lines of Mo^5+^ centers (Equation (2)). The error of the g tensor values determination was ±0.003. The error of the ΔH_pp_ determination was ±0.2 G. The error of the I determination was ±5 × 10^3^ arb. units.

Sample	Treatment	Mo Center	*g*_1_	*g*_2_	*g*_3_	ΔH_pp_, G	I, arb. Units
ZnO:Mo(20%) NRP	As grown	Mo1	1.918	1.918	1.918	40	70.23 × 10^5^
ZnO:Mo(20%) NRP	As grown	Mo2	1.938	1.925	1.890	20	11.70 × 10^5^
ZnO:Mo(30%) NRP	As grown	Mo1	1.918	1.918	1.918	40	119.38 × 10^5^
ZnO:Mo(30%) NRP	As grown	Mo2	1.938	1.925	1.890	20	198.95 × 10^5^
ZnO:Mo(10%) NRP	AA 350 °C	Mo1′	1.908	1.908	1.908	110	331.67 × 10^5^
ZnO:Mo(20%) NRP	AA 350 °C	Mo1′	1.908	1.908	1.908	110	21.77 × 10^5^
ZnO:Mo(20%) NRP	AA 350 °C	Mo2′	1.936	1.925	1.896	40	3.98 × 10^5^
ZnO:Mo(30%) NRP	AA 350 °C	Mo2′	1.936	1.925	1.896	40	8.85 × 10^5^
ZnO:Mo(30%) NRP	AA 350 °C	Mo2″	1.929	1.927	1.872	53	25.31 × 10^5^

## Data Availability

Not applicable.

## Supplementary Materials

The following supporting information can be downloaded at: https://www.mdpi.com/article/10.3390/ma16093294/s1, Figure S1: The XRD pattern of ZnO:Mo(10%) NRP shown along with the XRD patterns of ZnO, Zn_5_Mo_2_O_11_∙5H_2_O, C_12_H_7_NO_2_ and MoN PDF data (for details see main article); Figure S2: Mo^5+^ experimental EPR spectra measured at 60 K in the as grown ZnO:Mo(20%) (A) and ZnO:Mo(30%) (B) NRP samples shown along with the calculated signals. Mo3,4 indicate contributions from two different Mo^5+^ centers; Figure S3: Mo^5+^ experimental EPR spectra measured at 60 K after the annealing in air at 350 °C in the ZnO:Mo(10%) (A), ZnO:Mo(20%) (B) and ZnO:Mo(30%) (C) NRP samples shown along with the calculated signals. Mo1′,2′,2′′ indicate contributions from two different Mo^5+^ centers.

## References

[1] Grigorjeva L., Millers D., Smits K., Pankratov V., Łojkowski W., Fidelus J., Chudoba T., Bienkowski K., Monty C. (2009). Excitonic luminescence in ZnO nanopowders and ceramics. Opt. Mater..

[2] Uklein A., Multian V., Kuz’Micheva G., Linnik R., Lisnyak V., Popov A., Gayvoronsky V.Y. (2018). Nonlinear optical response of bulk ZnO crystals with different content of intrinsic defects. Opt. Mater..

[3] Zhang G.B., Zhou H.J., Shi C.S., Shi J.Y., Zhou Y.X., Zhang X.Y., Fu Z.X., Kirm M., Zimmerer G. (2002). Temperature and time dependence of emission properties of zno films deposited on si substrates. Surf. Rev. Lett..

[4] D’agostino D., Di Giorgio C., Bobba F., Di Trolio A., Alippi P., Cucolo A.M., Bonapasta A.A. (2019). Effects of cobalt substitution on ZnO surface reactivity and electronic structure. J. Mater. Chem. C.

[5] Kapat K., Shubhra Q.T.H., Zhou M., Leeuwenburgh S. (2020). Piezoelectric Nano-Biomaterials for Biomedicine and Tissue Regeneration. Adv. Funct. Mater..

[6] Siebert L., Luna-Cerón E., García-Rivera L.E., Oh J., Jang J., Rosas-Gómez D.A., Pérez-Gómez M.D., Maschkowitz G., Fickenscher H., Oceguera-Cuevas D. (2021). Light-Controlled Growth Factors Release on Tetrapodal ZnO-Incorporated 3D-Printed Hydrogels for Developing Smart Wound Scaffold. Adv. Funct. Mater..

[7] Leiter F., Zhou H., Henecker F., Hofstaetter A., Hofmann D., Meyer B. (2001). Magnetic resonance experiments on the green emission in undoped ZnO crystals. Phys. B Condens. Matter.

[8] Chen J.-X., Hao S.-T., Sun Z.-X., Zheng P., Tang J., Yang Y.-L., Zhang S.-L., Liu X.-L., Zhao J.-T., Li Q.-L. (2022). Development of the ZnO:Ga nanorod arrays as an alpha particle scintillation screen for the associated particle neutron generator. Appl. Phys. Lett..

[9] You D., Xu C., Wang X., Wang J., Su W., Wang R., Chen T., Shi Z. (2020). A core@dual-shell nanorod array with a cascading band configuration for enhanced photocatalytic properties and anti-photocorrosion. J. Mater. Chem. A.

[10] Zhou Y., Chen G., Sargent E.H., Zhuang T., Dinh C.T., He F. (2017). Freestanding nano-photoelectrode as a highly efficient and visible-light-driven photocatalyst for water-splitting. J. Mater. Chem. A.

[11] Ray C., Pal T. (2017). Retracted Article: Recent advances of metal–metal oxide nanocomposites and their tailored nanostructures in numerous catalytic applications. J. Mater. Chem. A.

[12] Barbillon G., Sandana V.E., Humbert C., Bélier B., Rogers D.J., Teherani F.H., Bove P., McClintock R., Razeghi M. (2017). Study of Au coated ZnO nanoarrays for surface enhanced Raman scattering chemical sensing. J. Mater. Chem. C.

[13] Feng H., Liang L., Wu W., Huang Z., Liu Y. (2020). Architecting epitaxial-lattice-mismatch-free (LMF) zinc oxide/bismuth oxyiodide nano-heterostructures for efficient photocatalysis. J. Mater. Chem. C.

[14] Garg N., White C.E. (2017). Mechanism of zinc oxide retardation in alkali-activated materials: An in situ X-ray pair distribution function investigation. J. Mater. Chem. A.

[15] Chen H., Shen K., Chen J., Chen X., Li Y. (2017). Hollow-ZIF-templated formation of a ZnO@C–N–Co core–shell nanostructure for highly efficient pollutant photodegradation. J. Mater. Chem. A.

[16] He G.-H., Jiang M.-M., Dong L., Zhang Z.-Z., Li B.-H., Shan C.-X., Shen D.-Z. (2017). Near-infrared light-emitting devices from individual heavily Ga-doped ZnO microwires. J. Mater. Chem. C.

[17] Buryi M., Babin V., Chang Y.-Y., Remeš Z., Mičová J., Šimek D. (2020). Influence of precursor age on defect states in ZnO nanorods. Appl. Surf. Sci..

[18] Neykova N., Hruska K., Holovsky J., Remes Z., Vanecek M. (2013). Arrays of ZnO nanocolumns for 3-dimensional very thin amorphous and microcrystalline silicon solar cells. Thin Solid Films.

[19] Mičová J., Buryi M., Šimek D., Drahokoupil J., Neykova N., Chang Y.-Y., Remeš Z., Pop-Georgievski O., Svoboda J., Im C. (2018). Synthesis of zinc oxide nanostructures and comparison of their crystal quality. Appl. Surf. Sci..

[20] Dujardin C., Auffray E., Bourret-Courchesne E., Dorenbos P., Lecoq P., Nikl M., Vasil’Ev A.N., Yoshikawa A., Zhu R.-Y. (2018). Needs, Trends, and Advances in Inorganic Scintillators. IEEE Trans. Nucl. Sci..

[21] Abrahams S.C., Bernstein J.L. (1969). Remeasurement of the structure of hexagonal ZnO. Acta Crystallogr. Sect. B Struct. Crystallogr. Cryst. Chem..

[22] Buryi M., Remeš Z., Babin V., Artemenko A., Vaněček V., Dragounová K.A., Landová L., Kučerková R., Mičová J. (2021). Transformation of free-standing ZnO nanorods upon Er doping. Appl. Surf. Sci..

[23] Buryi M., Remeš Z., Babin V., Novotný M., Vaněček V., Dragounová K.A., Mičová J., Landová L., Kučerková R., More-Chevalier J. (2021). Influence of Mo doping on the luminescence properties and defect states in ZnO nanorods. Comparison with ZnO:Mo thin films. Appl. Surf. Sci..

[24] Neykova N., Moulin E., Čampa A., Hruška K., Poruba A., Stuckelberger M., Haug F.-J., Topič M., Ballif C., Vanecek M. (2015). Three-dimensional amorphous silicon solar cells on periodically ordered ZnO nanocolumns. Phys. Status Solidi (A).

[25] Neykova N., Brož A., Remeš Z., Hruška K., Kalbáčová M., Kromka A., Vaněček M. (2012). ZnO hedgehog-like structures for control cell cultivation. Appl. Surf. Sci..

[26] Buryi M., Remeš Z., Babin V., Vaněček V., Dragounová K.A., Mičová J., Landová L., Kučerková R. (2021). ZnO nanorods alloyed with Mo/Er. The effect of post-deposition treatment on defect states and luminescence. IOP Conf. Ser. Mater. Sci. Eng..

[27] Buryi M., Remeš Z., Babin V., Chertopalov S., Děcká K., Dominec F., Mičová J., Neykova N. (2022). Free-Standing ZnO:Mo Nanorods Exposed to Hydrogen or Oxygen Plasma: Influence on the Intrinsic and Extrinsic Defect States. Materials.

[28] Buryi M., remeš Z., děcká K., Mičová J., Landová L. Transformation of ZnO-based structures under heavy Mo doping: Defect states and luminescence. Proceedings of the NANOCON 2021 Conference.

[29] Buryi M., Babin V., Artemenko A., Remeš Z., Děcká K., Mičová J. (2022). Hydrothermally grown ZnO:Mo nanorods exposed to X-ray: Luminescence and charge trapping phenomena. Appl. Surf. Sci..

[30] Neykova N., Kozak H., Ledinsky M., Kromka A. (2012). Novel plasma treatment in linear antenna microwave PECVD system. Vacuum.

[31] Buryi M., Remeš Z., Babin V., Artemenko A., Chertopalov S., Mičová J. (2022). Cold plasma treatment of ZnO:Er nano- and microrods: The effect on luminescence and defects creation. J. Alloys Compd..

[32] Buryi M., Ridzoňová K., Neykova N., Landová L., Hájek F., Babin V., Děcká K., Sharma R.K., Pop-Georgievski O. (2023). Effect of UV Irradiation on the Growth of ZnO:Er Nanorods and Their Intrinsic Defects. Chemosensors.

[33] Rajiv P., Dinnebier R.E., Jansen M. (2010). “Powder 3D Parametric”—A program for Automated Sequential and Parametric Rietveld Refinement Using Topas. Mater. Sci. Forum.

[34] Pop-Georgievski O., Kubies D., Zemek J., Neykova N., Demianchuk R., Chánová E.M., Šlouf M., Houska M., Rypacek F. (2015). Self-assembled anchor layers/polysaccharide coatings on titanium surfaces: A study of functionalization and stability. Beilstein J. Nanotechnol..

[35] Pop-Georgievski O., Neykova N., Proks V., Houdkova J., Ukraintsev E., Zemek J., Kromka A., Rypaček F. (2013). Polydopamine-modified nanocrystalline diamond thin films as a platform for bio-sensing applications. Thin Solid Films.

[36] Mooney J., Kambhampati P. (2013). Get the Basics Right: Jacobian Conversion of Wavelength and Energy Scales for Quantitative Analysis of Emission Spectra. J. Phys. Chem. Lett..

[37] Stoll S., Schweiger A. (2006). EasySpin, a comprehensive software package for spectral simulation and analysis in EPR. J. Magn. Reson..

[38] Nishinaga T. (2016). Organic Redox Systems: Synthesis, Properties, and Applications.

[39] Lo S.-S., Huang D., Tu C.-H., Jan D.-J. (2009). Formation and Raman scattering of seed-like ZnO nanostructure. J. Raman Spectrosc..

[40] Neykova N., Chang Y.-Y., Buryi M., Davydova M., Kucerkova R., Simek D., Remes Z., Pop-Georgievski O. (2019). Study of ZnO nanorods grown under UV irradiation. Appl. Surf. Sci..

[41] Li L.M., Li C.C., Zhang J., Du Z.F., Zou B.S., Yu H.C., Wang Y.G., Wang T.H. (2007). Bandgap narrowing and ethanol sensing properties of In-doped ZnO nanowires. Nanotechnology.

[42] Yang J.H., Zheng J.H., Zhai H.J., Yang L.L. (2008). Low temperature hydrothermal growth and optical properties of ZnO nanorods. Cryst. Res. Technol..

[43] Neykova N., Stuchlik J., Hruska K., Poruba A., Remes Z., Pop-Georgievski O. (2017). Study of the surface properties of ZnO nanocolumns used for thin-film solar cells. Beilstein J. Nanotechnol..

[44] Lyons J.L., Varley J.B., Steiauf D., Janotti A., Van de Walle C.G. (2017). First-principles characterization of native-defect-related optical transitions in ZnO. J. Appl. Phys..

[45] Frodason Y.K., Johansen K.M., Bjørheim T.S., Svensson B.G., Alkauskas A. (2017). Zn vacancy as a polaronic hole trap in ZnO. Phys. Rev. B.

[46] Thomas D. (1960). The exciton spectrum of zinc oxide. J. Phys. Chem. Solids.

[47] Meyer B.K., Alves H., Hofmann D.M., Kriegseis W., Forster D., Bertram F., Christen J., Hoffmann A., Straßburg M., Dworzak M. (2004). Bound exciton and donor–acceptor pair recombinations in ZnO. Phys. Status Solidi (B).

[48] Buryi M., Spassky D., Hybler J., Laguta V., Nikl M. (2015). Electron Spin Resonance study of charge trapping in α-ZnMoO4 single crystal scintillator. Opt. Mater..

[49] Spassky D., Nagirnyi V., Mikhailin V., Savon A., Belsky A., Laguta V., Buryi M., Galashov E., Shlegel V., Voronina I. (2013). Trap centers in molybdates. Opt. Mater..

[50] Teke A., Özgür Ü., Doğan S., Gu X., Morkoç H., Nemeth B., Nause J., Everitt H.O. (2004). Excitonic fine structure and recombination dynamics in single-crystalline ZnO. Phys. Rev. B.

[51] Wang Y.G., Lau S.P., Lee H.W., Yu S.F., Tay B.K., Zhang X.H., Hng H.H. (2003). Photoluminescence study of ZnO films prepared by thermal oxidation of Zn metallic films in air. J. Appl. Phys..

[52] Abragam A., Bleaney B. (2012). Electron Paramagnetic Resonance of Transition Ions.

[53] Jakes P., Erdem E. (2011). Finite size effects in ZnO nanoparticles: An electron paramagnetic resonance (EPR) analysis. Phys. Status Solidi (RRL)–Rapid Res. Lett..

[54] Anjana R., Jayaraj M.K., Yadav A.K., Jha S.N., Bhattacharyya D. (2018). Investigating the evolution of local structure around Er and Yb in ZnO:Er and ZnO:Er, Yb on annealing using X-ray absorption spectroscopy. J. Appl. Phys..

[55] Wang J., Zhou M.J., Hark S.K., Li Q., Tang D., Chu M.W., Chen C.H. (2006). Local electronic structure and luminescence properties of Er doped ZnO nanowires. Appl. Phys. Lett..

[56] Honglin L., Yingbo L., Jinzhu L., Ke Y. (2014). Experimental and first-principles studies of structural and optical properties of rare earth (RE = La, Er, Nd) doped ZnO. J. Alloy. Compd..

[57] Mackova A., Malinsky P., Pupikova H., Nekvindova P., Cajzl J., Svecova B., Oswald J., Wilhelm R., Kolitsch A. (2014). A comparison of the structural changes and optical properties of LiNbO_3_, Al_2_O_3_ and ZnO after Er+ ion implantation. Nucl. Instrum. Methods Phys. Res. Sect. B Beam Interact. Mater. Atoms.

[58] Van de Walle C.G. (2000). Hydrogen as a Cause of Doping in Zinc Oxide. Phys. Rev. Lett..

[59] Poole C.P., Farach H.A., American Institute of Physics (1999). Handbook of Electron Spin Resonance.

