# Supplementary material for: Changes to Material Phase and Morphology Due to High-Level Molybdenum Doping of ZnO Nanorods: Influence on Luminescence and Defects
Source: Materials (Basel). 2023 Apr 22;16(9):3294. doi: 10.3390/ma16093294 (PMC10178970; doi:10.3390/ma16093294)
Supplement: Supplementary file 1 [file materials-16-03294-s001.zip › materials-2305583-supplementary.pdf]

Supporting Information of the paper: "**Changes caused to material phase and morphology due to high-level molybdenum doping of ZnO nanorods: Influence on luminescence and defects**"

Maksym Buryi[1], Vladimir Babin[1*], Neda Neykova[1,2], Yu-Min Wang[3], Zdeněk Remeš[1], Katarína Ridzoňová[1,4], Filip Dominec[1], Marina Davydova[1], Jan Drahokoupil[1], Sergii Chertopalov[1], Lucie Landová[1,2], Ognen Pop-Georgievski[3]

[1]*FZU - Institute of Physics of the Czech Academy of Sciences, Na Slovance 1999/2, 182 21, Prague, Czechia*

[2]*Czech Technical University in Prague, Faculty of Electrical Engineering, Technická 2, 166 27 Prague, Czechia*

[3]*Department of Chemistry and Physics of Surfaces and Interfaces, Institute of Macromolecular Chemistry, Czech Academy of Sciences, Heyrovský sq. 2, 16206 Prague, Czechia*

[4]*Charles University, Faculty of Mathematics and Physics, Institute of Physics, Ke Karlovu 5, 121 16, Prague 2, Czechia*

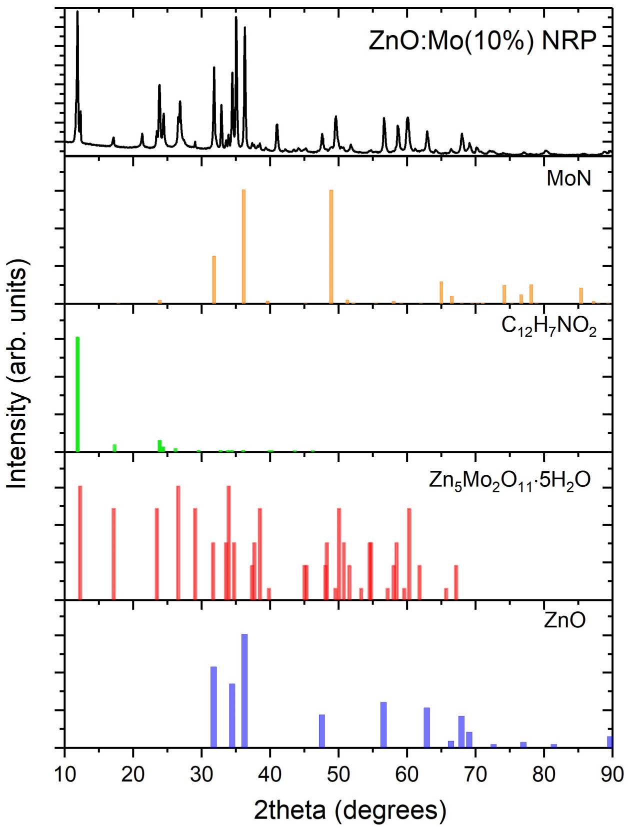

Figure S1 The XRD pattern of ZnO:Mo(10%) NRP shown along with the XRD patterns of ZnO, $Zn_5Mo_2O_{11}\cdot 5H_2O$, $C_{12}H_7NO_2$ and MoN PDF data (for details see main article).

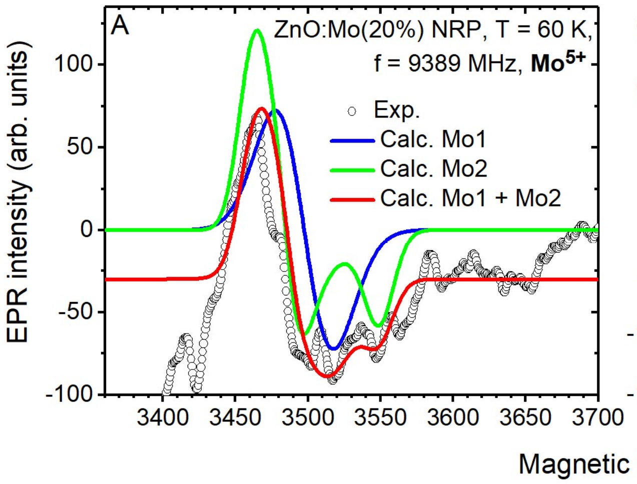

Figure S2 Mo^{5+} experimental EPR spectra measured at 60 K in the as grown ZnO:Mo(20%) (**A**) and ZnO:Mo(30%) (**B**) NRP samples shown along with the calculated signals. Mo3,4 indicate contributions from two different Mo^{5+} centers.

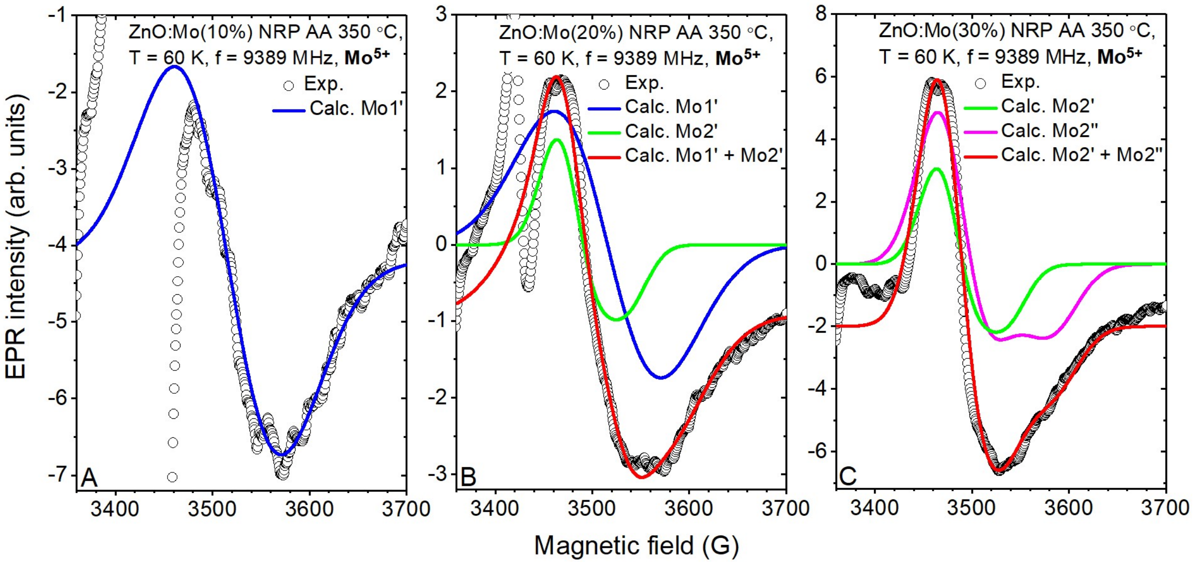

Figure S3 Mo^{5+} experimental EPR spectra measured at 60 K after the annealing in air at 350 °C in the ZnO:Mo(10%) (**A**), ZnO:Mo(20%) (**B**) and ZnO:Mo(30%) (**C**) NRP samples shown along with the calculated signals. Mo1′,2′,2″ indicate contributions from two different Mo^{5+} centers.
